# Editorial: Get Over the Gut: Apicomplexan Parasite Interaction, Survival and Stage Progression in Vertebrate and Invertebrate Digestive Tracts

**DOI:** 10.3389/fcimb.2021.680555

**Published:** 2021-04-28

**Authors:** Nicholas C. Smith, Robert E. Sinden, Chandra Ramakrishnan

**Affiliations:** ^1^ School of Life Sciences, University of Technology Sydney, Sydney, NSW, Australia; ^2^ Research School of Biology, Australian National University, Canberra, ACT, Australia; ^3^ Department of Life Sciences, Imperial College London, London, United Kingdom; ^4^ Institute of Parasitology, University of Zurich, Zurich, Switzerland

**Keywords:** Apicomplexa, barrier, gut, immune system, microbiota, host, model system

## Introduction

For endoparasites, invasion of their hosts represents the greatest challenge to survival; for many it is the gut of the host/vector that present this barrier. Those barriers are increasingly being recognized as optimal targets for intervention strategies (Sinden, 2010; Smith et al., 2014; Sinden, 2017). The phylum Apicomplexa embraces thousands of species of parasites of vertebrates and invertebrates, many of major veterinary/medical importance including important agents of zoonoses. Whilst certain parasites are monoxenous (*e.g.*, *Eimeria*, *Cryptosporidium*), others are heteroxenous (*e.g. Plasmodium*, *Toxoplasma gondii*) with distinct developmental pathways in each host. All Apicomplexans are thought to undergo critical developmental phases within intestinal tracts; thus, infection or transition of a gut is crucial for their survival. In this Research Topic, we present contributions on mechanisms of gut infection and traversal; the gut as a barrier to parasites; model systems for parasite development, and the impact of gut microbiota upon the infection process.

## The Gut: An Entrance and a Barrier

The gut is a parasite entry point, but also a significant host barrier to infection. In the case of *T. gondii* infecting intermediate hosts, the gut offers several routes by which to reach the lamina propria and beyond: direct infection of epithelial cells and replication before moving further; intercellular traversal; or subversion of immune cells to become ‘Trojan horses’. These processes trigger immune responses, and cells serving as Trojan horses can also be involved in combatting parasites (reviewed by Snyder and Denkers). Additionally, whilst parasites may need to protect themselves from the complement system, there may be an advantage if they stimulate a certain level of protective immunity to avoid killing their hosts *via* unchecked proliferation (reviewed in Sikorski et al.). Moreover, host and parasite are rarely alone; the ‘environmental’ microbiota play an important role in regulation of immunity and inflammation (reviewed by Snyder and Denkers and Sikorski et al.).

*Plasmodium* encounters the gut after having been taken up by the mosquito in a blood meal. Gametogenesis and fertilization happen rapidly after arrival in the midgut, being triggered by the changes in environment. The midgut lumen is a hostile place for *Plasmodium*, with factors from both the blood and the mosquito targeting the parasite. To escape this attack, the fertilized parasite transforms into a motile ookinete, which immediately faces the obstacle of a chitin-rich peritrophic matrix. To overcome this barrier, *Plasmodium* is thought to use a complex comprised of proteins that mediate recognition and attachment and a chitinase to digest chitin (Patra et al.). Additionally, mitochondrial function of the midgut epithelial cells is dynamic and thought to be involved in resistance to pathogens (reviewed in Luckhart and Riehle).

Using loss-of-function mutants, a large number of genes involved in ookinete motility, invasion and differentiation have been identified in *Plasmodium*. However, not only the absence or presence of a gene, but the fine-tuning of its expression may play a role in the parasite’s capacity to overcome the gut and differentiate. Witmer et al. hypothesize that genes may be differentially regulated among different individual ookinetes as a potential ‘bet-hedging’ strategy. Using single-cell RNA sequencing, they show that transcriptional variations occur within inbred populations and propose candidate genes involved in bet-hedging. Once the parasite gets to the other side of the midgut, differentiation to oocysts takes place; Ukegbu et al. have identified three genes that are important in the transition of ookinetes to oocysts, likely operating downstream of midgut traversal.

## Model Systems to Study Parasite-Gut Interaction

Studying the interplay between parasite and the gut is not straightforward because either *in vitro* systems or *in vivo* models have not yet been established or do not support all stages of development. *In vitro* systems are also commonly a prerequisite for high through-put screening of anti-parasitic drugs and may be useful for the identification of vaccine candidates.

In the case of a major agent of chicken coccidiosis, *Eimeria tenella*, *in vitro* systems only support the development of early asexual stages or produce very low yields of sexual stages and oocysts. However, innovative use of Madin-Darby Bovine Kidney (MDBK) epithelial cells has enabled researchers to mimic sporozoite invasion *in vitro* and even obtain first generation merozoites (Marugan-Hernandez et al., 2020). Additionally, this *in vitro* platform enables screening of anticoccidial drugs. Several *in vitro* platforms using multiple cell types have shown that sexual development of coccidia may be better supported in such systems (Chen et al., 2013; Worliczek et al., 2013; Martorelli Di Genova et al., 2019). The situation is similar for *Cryptosporidium*, for which two- and three-dimensional cell culture systems supporting sexual development have been established recently (reviewed by Marzook and Sateriale). Additionally, an *in vivo* mouse model for human cryptosporidiosis, using the genetically tractable *Cryptosporidium tyzzeri*, is now available (reviewed by Marzook and Sateriale). Holthaus et al. have achieved harmonized protocols for cultures of human, mouse, pig and chicken intestinal cells that are important hosts for protozoan infections. Using the example of *T. gondii* tachyzoites and *Giardia duodenalis*, the authors also demonstrate the potential utility of their system for co-infection studies.

All these *in vitro* platforms utilize complex media and cellular environments but, whilst more sophisticated than monolayer cultures, they lack important players in the interaction of the host with the parasite, namely, immune cells and microbiota. Introducing bacteria and fungi into these systems is desirable because experiments without microbiota may lead to conclusions that are insecure, as exemplified by a recent *in vivo* study on *E. tenella* showing that, in the absence of intestinal microbiota, parasite replication is altered (Gaboriaud et al.). However, developing *in vitro* systems that incorporate microbiota is challenging because these systems require more complex media to support the growth of diverse cell types, and lack important host factors that control/suppress growth of the replicating bacteria/fungi.

## Perspectives

This Research Topic, with examples from four genera of Apicomplexa, documents the innovative research currently being performed to address interactions between gut and parasite and highlights the fact that the interplay between the two is complex, with both parties having well-developed strategies for their own survival. Novel tools and methodologies are being developed, state-of-the-art techniques expanded, and many new insights gained. As knowledge gaps are being closed, doors are being opened to develop novel intervention strategies against these parasites.

## Author Contributions

NS, RS and CR have written the editorial. All authors contributed to the article and approved the submitted version.

## Funding

NS is grateful for research support from the National Health and Medical Research Council (NHMRC) of the Australian Government (Project Grant GNT1128911). These funders played no role in interpretation of data and information researched for this article, its writing, or the decision to submit it for publication.

## Conflict of Interest

The authors declare that the research was conducted in the absence of any commercial or financial relationships that could be construed as a potential conflict of interest.
